# Dispatch in disasters - a descriptive analysis of pre-hospital surge capacity in Rwanda’s Emergency medical services

**DOI:** 10.1016/j.afjem.2026.100955

**Published:** 2026-03-06

**Authors:** Lotta Velin, Eric Mugabo, Laura Pompermaier, Jeanne d’Arc Nyinawankusi, Andreas Wladis, Menelas Nkeshimana

**Affiliations:** a Center for Disaster Medicine and Traumatology (KMC), Department of Biomedical and Clinical Sciences, Linköping University, Linköping, Sweden; b OAZIS Health, Kigali, Rwanda; c Department of Planning, Health Financing, Monitoring and Evaluation, Ministry of Health, Kigali, Rwanda; d Division of Emergency Medical Services, Rwanda Biomedical Center, Kigali, Rwanda; e Centre Hospitalier Universitaire de Kigali, Department of Internal Medicine, Kigali, Rwanda; f Department of Health Workforce Development, Ministry of Health, Kigali, Rwanda

**Keywords:** Trauma care, Surge capacity, Emergency medical services, Low-resource setting, Eastern Africa, Mass casualty incident, Disaster

## Abstract

**Background:**

Emergency medical services (EMS) are essential to mass casualty incident (MCI) response, yet there is limited data from low-resource settings, where trauma is most prevalent. In Rwanda, trauma is the most common case seen by EMS, but the burden of MCIs remains unclear. This study aimed to describe EMS response to MCIs in Rwanda and to identify factors influencing ambulance dispatch.

**Methods:**

A retrospective analysis was conducted using the Rwandan EMS call centre registry from July 1, 2018, to September 1, 2020. MCIs were defined as incidents with ≥3 injured individuals. Univariate analysis assessed MCI occurrence and ambulance dispatch by location (urban/rural) and time (day/night). Multivariate logistic and linear regressions examined predictors of ambulance dispatch and the number of ambulances sent. Results are presented as odds ratios (OR) and regression coefficients with 95% confidence intervals.

**Results:**

Of 18,059 entries, 9019 (49.9%) involved trauma. In total, 173 MCIs were described, including 770 injured individuals. Most MCIs occurred in Kigali (67.1%), and were road traffic accidents (96.0%). The median number of casualties per MCI was 3 (IQR 3–4). Injuries were mainly orthopaedic (65.3%) and moderate (46.2%). Ambulances were dispatched from the central command post in 67.7% of cases, whereas emergency teams were dispatched from nearby facilities in nearly 1/3. Most patients were transported to district hospitals. EMS dispatch was significantly more likely in Kigali (OR=60.4, *p* < 0.01), but not influenced by casualty count (OR=0.92, *p* = 0.36). However, higher casualty numbers did predict more ambulances sent (*p* < 0.01).

**Conclusion:**

Rwanda experiences approximately 6.7 MCIs monthly across all healthcare levels. EMS dispatch was more likely in Kigali, though the number of ambulances correlated with the casualty count. Future research should explore the mortality knowledge gap and pre-hospital surge strategies, including EMS-hospital team collaboration, multi-patient transport, and on-site treatment of minor injuries.

## African Relevance

• Mass casualty incidents, especially due to road traffic accidents, are common in Africa
• Emergency medical services (EMS) play a key role in mass-casualty management
• EMS are lacking or inadequate in many parts of Africa
• Rwanda has one of the leading EMS in Africa, with nationwide coverage; however, little is known about pre-hospital surge capacity in Rwanda or in Africa
• This is a retrospective study assessing EMS response to MCIs in Rwanda and identifying factors influencing ambulance dispatch
• The study findings may have generalizability to comparable EMS systems across the continent and provides suggestions for contextually relevant pre-hospital surge strategies to increase capacity

## Introduction

Mass casualty incidents (MCIs) challenge health systems by causing patient surges. Emergency medical services (EMS) are the first point of contact between severely injured patients and the health system, responsible for field triage, initial stabilisation, basic life support, and transport to definitive care. EMS also provides essential situational reports to coordinate the response. However, MCI understanding is limited by challenges linking patients to incidents and by missing prehospital data [[1], [2], [3]]. There is also a survivorship bias, where only patients who reach the hospital alive are typically included in trauma registries.

Most MCI research comes from high-income countries with uncertain generalizability to low-resource settings where EMS are nonexistent or limited [4]. Limited coverage disproportionally affects rural areas, where private transport is common. Rwanda has made major road safety improvements, such as mandating helmets for motorcycle riders and seat belts for car passengers [5,6], yet, as in many low-resource settings [7], road traffic accidents remain common [8], often occurring as MCI [9,10]. Although Rwanda’s EMS aims to provide nationwide coverage, operation density is highest in the capital city, Kigali, indicating a possible rural knowledge gap [11].

This cross-sectional study aimed to describe EMS response to MCIs in Rwanda and to identify factors influencing ambulance dispatch.

## Methods

### Study context

Rwanda is a densely populated low-income country of about 26,000 km² with over 13 million inhabitants. The referral-based health system includes tertiary hospitals and specialist care, mainly concentrated in urban areas. Nearly the entire population is covered by *Mutuelle de Santé*, a community-based health insurance scheme supporting universal health coverage [12,13]. The country’s mountainous terrain and limited rural infrastructure pose challenges for EMS dispatch [14]. The national EMS, previously called SAMU (Service d’Aide Medical d’Urgence), has operated since 2007 [11]. When a traumatic event occurs, bystanders call a toll-free number to a 24/7 call centre. If considered an emergency, EMS is dispatched [15]. Ambulance teams consist of a driver and two nurses, or one nurse and one non-physician anaesthetist in more critical cases. Depending on the initial assessment, additional EMS teams may be dispatched. All staff are trained in basic life support (BLS), with current plans to provide advanced life support (ALS) training.

The call centre registry is completed using information from first responders and supplemented by the ambulance crew. It includes variables on event location and time, first responder information, event type, initial assessment, and injury severity (“minor”, “moderate”, and “severe”), ambulance dispatch (yes/no, and if “yes”, the number of EMS teams), and patient management (on-site or transfer). Generally, one entry corresponds to one patient. Individuals found dead are not transported by EMS, but are recorded if possible. No structured field triage was used at the time of the study. However, EMS used checklists for extremity injury, head injury, and burn assessment [16], and these recommend a general “ABC” approach [15,17].

### Data source

Data from the EMS call centre registry from July 1st, 2018, until September 1st, 2020, were obtained. Entries describing non-traumatic events were excluded.

### Data analysis

All trauma entries were assessed to detect likely MCIs. The variables “event description" (free text) and “initial assessment” (injury descriptions) were reviewed. Entries mentioning ≥3 injured patients from one incident needing treatment were coded as “MCI”. The threshold was chosen based on Rwandan expert consensus, as there is no globally accepted MCI definition. Additionally, entries from the same location (identical “district”, “sector”, and “cell”) within one hour, with consistent trauma mechanisms, were coded as a single MCI.

After screening, the unit of analysis changed from patients to events by grouping patients by incident. This dataset was used for descriptive statistics of MCIs, including location, timing, mechanism, number of injured and deceased, injury type, ambulances dispatched, other response teams, patient transfers, and destination facilities. Injuries were grouped under major anatomic injury classification headings. Because prior studies suggest most MCIs occur during evenings or nights [18], a “night” variable was generated from 5 PM to 8 AM, which is the most common night shift slot in Rwanda. A separate variable was created for 11 PM to 5 AM, as these represent hours when calling in staff may be especially challenging.

Data normality was tested using the Shapiro-Wilk test. Descriptive statistics (mean, standard deviation (SD), median, and interquartile range) were reported, along with frequencies and percentages. Fisher’s exact test compared categorical variables (Kigali vs non-Kigali; night vs day). The Student *t*-test compared numeric variables (number of injured, on-site deaths, and ambulances).

Regression analysis assessed confounders in ambulance dispatch. Odds ratios (OR) and regression coefficients were presented with 95 % confidence intervals (CI). StataBE 18.5 was used for analysis, with two-sided p-values <0.05 considered significant.

The Rwanda National Ethics Committee (76/RNEC/2024) gave ethical approval on April 2nd, 2024. The Director General of the Rwanda Biomedical Centre provided written approval to the dataset request. The study was conducted according to the RECOD checklist.

## Results

### Trauma events

**Fig. 1:**
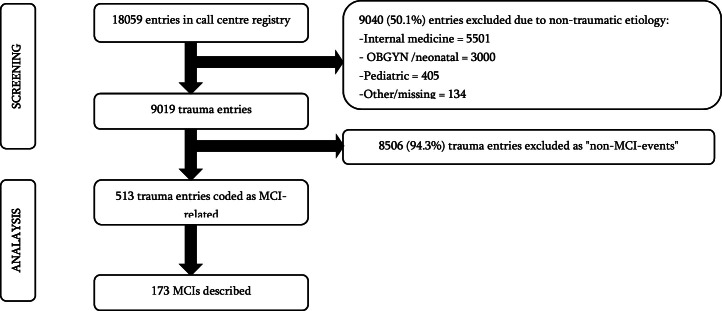
A flow chart describing the identification of MCIs amongst all call centre registry data.

Of the 18,059 entries in the call centre registry, 9019 entries (49.9 %) were categorised as trauma with appropriate circumstances described (Fig. 1). Amongst the 9019 trauma entries, 173 MCIs were described. This corresponds to an average of 6.7 MCIs per month and an incidence of 0.63 MCIs per 100,000 inhabitants per year. The MCIs involved 770 injured individuals, and 21 were described as dead on-site. Most MCIs occurred in Kigali (*n* = 116, 67.1 %), followed by the Southern Province (*n* = 25, 14.5 %) and the Northern Province (*n* = 14, 8.1 %). The proportion of MCIs amongst all trauma-related data entries was significantly higher amongst entries from outside Kigali than amongst entries from Kigali (9.7 % vs 5.2 %, *p* < 0.01).

Most commonly, MCIs were road traffic accidents (*n* = 166, 96.0 %; Table 1). Most RTAs involved a car or bus (*n* = 100, 57.8 %), followed by motorcycles (*n* = 84, 48.6 %), pedestrians (*n* = 35, 20.2 %), and bicycles (*n* = 18, 10.4 %). Most MCIs (*n* = 99, 57.2 %) occurred in the night-time (5 PM – 8 AM), but only 13.3 % (*n* = 23) occurred between 11 PM and 5 AM.

**Table 1:** An overview of the demographic characteristics of patients from MCIs.

	N	%
**Mass Casualty Incident Type**		
Road traffic accidents	166	96.0
Burn/blast	5	2.9
Assault/Aggression	1	0.6
Landslides	1	0.6
**Geographical Location**		
Kigali	116	67.1
Outside of Kigali	57	33.0
**Type Of Injury**		
Orthopaedic injury	112	64.7
Abrasions / Soft tissue / burns	104	60.1
Head injury	71	41.0
Maxillofacial/orbital injury	51	29.5
Abdominal or thoracic injury	45	26.0
Altered mental status / coma	14	8.1

The median number of injured patients per MCI was 3 (IQR 3–4, range 3–32). In 10 MCIs, at least one patient was found dead on-site, with the number of on-site deaths reported ranging from 1–4.

### Injury characteristics

Injuries were most commonly orthopaedic (*n* = 113, 65.3 %), followed closely by soft tissue injuries (*n* = 104, 60.1 %). Amongst orthopaedic injuries, fractures were the most frequent (*n* = 91, 52.6 %), most commonly involving lower limbs (*n* = 65, 37.6 %). Combined tibia and fibula fractures (*n* = 31) was the leading fracture type, followed by femur fractures (*n* = 22). In 40 MCIs, open fractures were described (44.0 % of all fractures).

According to the EMS injury severity classification, nearly half of the entries were labelled as moderate (*n* = 237, 46.2 %), followed by minor (*n* = 146, 28.9 %) and severe (*n* = 128, 25.0 %).

### Ambulance dispatch

In 67.7 % (*n* = 115) of the incidents, EMS intervened through one or several teams from the call centre. In Kigali, EMS teams were dispatched in 93.0 % of MCIs (*n* = 106/114), compared to in only 16.1 % of MCIs outside Kigali (*n* = 9/56), *p* < 0.01. Additionally, the number of injured patients was higher in non-Kigali MCIs with EMS intervention (mean 6.2 injured versus 3.7 in Kigali (*p* < 0.01)). In 43.6 % (*n* = 51) of MCIs, only one ambulance was dispatched, and in 50 incidents (42.7 %), two ambulances were dispatched. There was no significant difference in EMS intervention in the night-time (*n* = 49/73, 67.1 %) versus day-time (*n* = 66/97, 68.0 %, *p* = 1.00), or in the sub-set-analysis with the 11 PM - 5 AM night-variable (*n* = 15/22, 68.2 %, *p* = 1.00).

In total, 202 EMS teams were dispatched in 115 MCIs, with a median of 2 teams per MCI (IQR 1–2), transporting 391 patients. This implied a mean of 1.94 patients per ambulance.

In nearly 1/3 of MCIs (*n* = 52, 30.1 %), emergency teams were dispatched from nearby hospitals, instead of formal EMS. In three MCIs (1.7 %), these teams came from multiple hospitals. In five events (2.9 %), EMS and local hospital teams were both present. In two events, patients used private transport to reach health facilities. No boat or helicopter ambulance transport was reported.

### Ambulance transport for further care

Some 760 patients were transferred for further care. Most commonly, patients were taken to district hospitals, which were the destination facility for at least one patient in 61.9 % of MCIs (*n* = 107), followed by at least one patient being brought to a level 1 hospital in 47.4 % (*n* = 82) of MCIs. In only eight MCIs (4.6 %), patients were taken to level 2 hospitals. The most common destination was Kibagabaga District Hospital (*n* = 64), followed by University Teaching Hospital of Kigali (CHUK) (*n* = 62) and Rwanda Military Referral and Teaching Hospital (*n* = 16). In 32 MCIs, at least one patient was taken to a health centre. In 59 MCIs, patients were distributed to multiple health facilities.

In nine incidents (5.3 %), patients were treated on-site only. The mean number of injured patients in these incidents was 5.2 (SD 1.5). The injuries managed on-site were: a traumatic lip wound, one suspected closed finger fracture, four abrasions, one hand laceration, and one with back pain after a road accident. In two events, four dead persons were managed on-site only, although it is not clear whether there were any resuscitation efforts or whether they were found dead upon arrival.

### Multivariate regression

Model 1 (geographic location only) and 3 (geographic location and night-time) had an R^2^ of 0.5, compared with the univariate model and Model 2 (night-time only), which had an R^2^ of 0.0. Model 3 was chosen as the final model (Table 2). In the final model, the number of injured persons was not significantly associated with EMS intervention (OR=0.92, *p* = 0.36) after adjusting for location (Kigali vs non-Kigali, OR=60.4, *p* < 0.01) and night-time (OR=0.89, *p* = 0.83).

**Table 2:** The final model for the logistic regression (EMS intervention (dichotomized as “yes/no”) as the dependent variable).

Variable	Odds ratio (95 % CI)	P-value
Number of injured persons	0.92 (0.77–1.1)	0.362
Kigali location	60.4 (21.5–169.5)	<0.01
Night-time	0.89 (0.32–2.50)	0.826

In the linear regression, the R^2^ was the same (0.16) for models 1 and 3, and slightly lower for model 2 (0.15). The number of injured persons was the only significant predictor of the number of ambulances dispatched (*p* < 0.01, Table 3).

**Table 3:** The final model for the linear regression (the number of EMS ambulances dispatched as the dependent variable).

Variable	Regression co-efficient (95 % CI)	P-value
Number of injured persons	0.23 (0.11–0.31)	<0.01
Kigali location	0.25 (−0.26–0.27)	0.34
Night-time	0.0040 (−0.26- 1.3)	0.98

## Discussion

This study found a significantly higher likelihood of EMS dispatch in Kigali-based MCIs compared with rural settings. Such an urban-rural divide is well-documented across sub-Saharan Africa at large [19], where only 8.7 % of the population has EMS coverage [4]. Similar disparities exist in high-income countries, where rural areas see longer response times [20]. The 2018 Rwandan National Surgical, Obstetric and Anaesthesia Plan (NSOAP) set a goal of 100 % two-hour ambulance access nationwide and one-hour access for major trauma by 2024 [21]. In rural areas, local hospital emergency teams are often dispatched due to proximity to ensure a timely response. Integrating these local teams within the national EMS system may strengthen surge capacity, and improvement of coordination between health facilities and EMS is a goal outlined by the Ministry of Health [14]. However, these hospital-based teams are rarely dedicated solely to pre-hospital care and face challenges with dispatch coordination, limited supplies, and insufficient training [14]. Future studies should assess whether the care quality is comparable to that of central EMS teams.

Rwanda’s health system manages a mean of 6.7 MCIs per month, reflecting high exposure across facility levels. This is comparable to a Malawian study [9] reporting in five months, also using a threshold of three injured. However, that was a single-centre study, in a referral hospital with approximately 600 beds (comparable with the second most common facility referral in our study: the University Teaching Hospital of Kigali). Hence, the overall MCI incidence is likely higher in Malawi. Notably, Kibagabaga Hospital, then a district hospital, was the most frequent MCI referral site, possibly due to its central Kigali location and greater bed availability compared with overcrowded referral hospitals.. It has since then been upgraded to a level 2 teaching hospital. These findings suggest that strengthening trauma care and surge capacity should extend beyond the level 1-hospitals.

Similar to previous studies from Nigeria, Malawi, and Haiti [9,22,23], and to a lesser degree studies from high-income countries [2,24], most MCIs in this study were road traffic accidents. However, the MCIs in this study involved fewer patients than the MCIs described in Malawi and Nigeria, where the mean number of injured persons was 8.4 and 13.1, respectively, despite the Malawi study using the same MCI threshold. The injuries described in Malawi were predominantly minor, including abrasions (31.9 %) and lacerations (30.6 %) [9], and in Nigeria, abrasions were also most common (in 61 % of patients) [23]. However, in the Nigerian study, over a quarter (*n* = 62/236, 26 %) were brought in dead. This combination of high mortality and predominantly minor injuries reaching the hospitals likely reflects survivorship bias. This contrasts with our study, where fractures were the most common injury, and head injuries were reported in over a third of MCIs; such injuries were less common in Malawi, with proportions of 14.3 % and 7.5 %, respectively. These discrepancies may also reflect Rwanda’s wide EMS coverage and recent road safety policy measures, such as the introduction of speed governors, speed limit radars, obligatory helmets, and a zero drunk-driving policy [5]. However, the mortality in this study must be viewed with caution, as it also likely contains survivorship bias, with on-site deaths not routinely reported, preventing a comprehensive understanding of MCI mortality.

This study highlights potential surge strategies to increase pre-hospital capacity. In Europe and the U.S., one patient per ambulance is the norm [25]. However, this study finds that patients with minor injuries are sometimes co-transported during MCIs. Whether this strategy has negative consequences on patient care, including conflicts with patient integrity and privacy, should be assessed in future studies.

In some MCIs, as well as in other trauma events, patients were treated on-site rather than referred, mainly for soft tissue injuries such as abrasions and lacerations, or simple procedures like wound suturing. Avoiding referrals for minor injuries aligns with field triage guidelines [26] and MCI triage systems like SALT, which recommend that “walking and talking” patients should not be brought to major hospitals, to prevent overcrowding and overburdening of the staff and resources [27]. However, given limited EMS resources, it is debatable whether on-site treatment increases ambulance waiting times. Pre-hospital management generally follows a ‘stay and play’ or ‘load and go’ approach, depending on whether early initial treatment or rapid transfer is prioritized. In less mature trauma systems, with limited EMS capabilities, longer pre-hospital times have been associated with worse outcomes [28,29]. However, since on-site treatment in this study mostly involved minor injuries, it may help reduce the burden on potentially overwhelmed health facilities. Yet, the risk of missed or delayed diagnosis, and legal pitfalls should be assessed in future studies before considering wider implementation.

Another pre-hospital surge capacity strategy is patient distribution to multiple health facilities. However, triage for lower-level facilities must be done carefully. Studies from India and South Africa found that critically injured patients first taken to lower-level facilities had nearly double the mortality risk (OR 1.9, *p* < 0.01) compared with those transported directly to tertiary hospitals, even after adjusting for injury severity and other patient factors [30].

### Limitations

The number of MCIs described is likely an understatement, resulting from multiple bias sources. Firstly, there may be entries falsely labelled as “non-MCI”, due to unclear reporting, since the data were not primarily collected for research purposes. Secondly, the registry data only reflect patients seen by EMS, although private transport is also frequent [28,30]. However, Rwanda’s EMS is well-established compared to many other low-resource settings [19], and the selection bias is hence likely to be lower in this study. Due to a lack of standardised reporting, this study cannot assess mortality, which limits a comprehensive understanding of MCIs.

The threshold of three or more injured patients was made at the incident level. However, in well-organised systems, as demonstrated in this study, patients may be distributed to different health facilities, mitigating a patient surge in a single facility. It could be argued that the MCI definition should be made at the facility level; however, we believe that, for this study, which aimed to assess the pre-hospital surge capacity, it was more appropriate to consider the entire incident.

Analysis of injury type, severity, and the number of critically injured patients in the MCIs is restricted by data uncertainty. Some diagnoses, which are difficult to determine clinically, such as closed fractures, are provided in the injury descriptions. The information provided was used for analysis without any further validation measures to ensure a correct diagnosis. Furthermore, the variable “intervention severity” reflects a subjective decision without any specific criteria, with cases such as open fractures, chest trauma, and traumatic brain injury being labelled as minor injuries in some cases. No specific injury coding system, such as ICD codes, is used in the registry, which could be an area for improvement to facilitate data management for quality control and research. Finally, to adequately evaluate pre-hospital MCI management, assessment of dispatch time, response time, and total pre-hospital time would be useful. An analysis of EMS transport time in 2018 found the mean total prehospital time for any emergency in Kigali to be 59 min, with 39 % exceeding 60 min [31]. Whether this can be extrapolated to MCI scenarios remains uncertain, but rural areas and MCI scenarios are likely associated with delays in pre-hospital response.

## Conclusion

Rwanda has a high MCI exposure, with a pre-hospital ambulance dispatch system with good coverage in Kigali, but limitations in rural areas. However, this is often mitigated by EMS teams from nearby health facilities. The number of injured persons was the main determinant for the number of ambulances dispatched, regardless of geographic location. Pre-hospital surge strategies may help increase capacity during MCIs, but should be assessed together with survivorship bias in future studies. Expansion of EMS should be prioritized, especially in rural areas, and formalized EMS protocols should be developed.

## Dissemination of results

The study team plans to present the published results to the Emergency Medical Services and the Ministry of Health. Rwandan team members will present the results at national and local conferences.

## CRediT authorship contribution statement

**Lotta Velin:** Conceptualization, Methodology, Software, Validation, Formal analysis, Investigation, Resources, Data curation, Writing – original draft, Visualization, Project administration. **Eric Mugabo:** Methodology, Investigation, Writing – review & editing. **Laura Pompermaier:** Conceptualization, Methodology, Resources, Writing – review & editing. **Jeanne d’Arc Nyinawankusi:** Conceptualization, Writing – review & editing. **Andreas Wladis:** Conceptualization, Writing – review & editing. **Menelas Nkeshimana:** Conceptualization, Methodology, Supervision, Writing – review & editing.

## Declaration of competing interest

There are no conflicts of interest to declare.
